# Supplementary material for: Extracellular Vesicle Abundance, but Not a High Aggregation-Prone Peptide Cargo, Is Associated with Dihydroartemisinin Exposure in Plasmodium falciparum
Source: Int J Mol Sci. 2025 Apr 22;26(9):3962. doi: 10.3390/ijms26093962 (PMC12072043; doi:10.3390/ijms26093962)
Supplement: Supplementary file 1 [file ijms-26-03962-s001.zip › supplementary/1_Supplementary.pdf]

**Supplementary Table S1. Summary data of EVs extraction experiment for all parasite lines and treatment conditions.**

| parasite | condition | replicate | sensitivity | parasitemia | mean_kcps | duration | kc_count | df1 | df2 | df3 | kc_total | kc_norm | EV size (nm) | Intensity/100 | EVs_count (x10$^{6}$) |
|---|---|---|---|---|---|---|---|---|---|---|---|---|---|---|---|
| PfPfVps60_KO | untreated | 1 | sensitive | 4.2 | 147.9 | 80 | 11832 | 0.25 | 0.3 | 0.1 | 1577600 | 375619.0476 | 213.4 | 0.945 | 354.96 |
| PfVps60_KO | untreated | 2 | sensitive | 4.4 | 117.8 | 90 | 10602 | 0.25 | 0.3 | 0.1 | 1413600 | 321272.7273 | 192 | 1 | 321.2727273 |
| PfVps60_KO | untreated | 3 | sensitive | 4.5 | 137.4 | 80 | 10992 | 0.5 | 0.3 | 0.1 | 732800 | 162844.4444 | 205.4 | 0.984 | 160.2389333 |
| PfVps60_KO | DMSO | 1 | sensitive | 4.1 | 19.9 | 130 | 2587 | 0.2 | 0.3 | 0.1 | 431166.667 | 105162.6016 | 281.8 | 0.838 | 88.12626016 |
| PfVps60_KO | DMSO | 2 | sensitive | 4.3 | 25.2 | 240 | 6048 | 0.25 | 0.3 | 0.1 | 806400 | 187534.8837 | 160.3 | 0.723 | 135.5877209 |
| PfVps60_KO | DMSO | 3 | sensitive | 4.4 | 24.8 | 170 | 4216 | 0.25 | 0.3 | 0.1 | 562133.333 | 127757.5758 | 179.8 | 0.728 | 93.00751515 |
| PfVps60_KO | DHA | 1 | sensitive | 0.2 | 93 | 50 | 4650 | 0.05 | 0.3 | 0.1 | 3100000 | 3100000 | 267.6 | 0.507 | 1571.7 |
| PfVps60_KO | DHA | 2 | sensitive | 0.1 | 93.5 | 90 | 8415 | 0.05 | 0.3 | 0.1 | 5610000 | 5610000 | 170.7 | 0.88 | 4936.8 |
| PfVps60_KO | DHA | 3 | sensitive | 0.2 | 39.3 | 180 | 7074 | 0.2 | 0.3 | 0.1 | 1179000 | 1179000 | 228.5 | 0.74 | 872.46 |
| 3D7 | untreated | 1 | sensitive | 2.4 | 183.4 | 70 | 12838 | 0.25 | 0.3 | 0.1 | 1711733.33 | 713222.2222 | 213.5 | 1 | 713.2222222 |
| 3D7 | untreated | 2 | sensitive | 2.5 | 82.7 | 100 | 8270 | 0.35 | 0.3 | 0.1 | 787619.048 | 315047.619 | 225 | 0.96 | 302.4457143 |
| 3D7 | untreated | 3 | sensitive | 2.1 | 149.6 | 80 | 11968 | 0.45 | 0.3 | 0.1 | 886518.519 | 422151.6755 | 202.6 | 0.981 | 414.1307937 |
| 3D7 | DMSO | 1 | sensitive | 2.2 | 29.7 | 100 | 2970 | 0.1 | 0.3 | 0.1 | 990000 | 450000 | 215.6 | 0.889 | 400.05 |
| 3D7 | DMSO | 2 | sensitive | 2.3 | 117.9 | 100 | 11790 | 0.5 | 0.3 | 0.1 | 786000 | 341739.1304 | 212 | 0.988 | 337.6382609 |
| 3D7 | DMSO | 3 | sensitive | 2.2 | 219 | 50 | 10950 | 0.1 | 0.3 | 0.1 | 3650000 | 1659090.909 | 139.7 | 1 | 1659.090909 |
| 3D7 | DHA | 1 | sensitive | 0.5 | 49.9 | 140 | 6986 | 0.3 | 0.3 | 0.1 | 776222.222 | 776222.2222 | 225 | 0.88 | 683.0755556 |
| 3D7 | DHA | 2 | sensitive | 0.6 | 74.9 | 90 | 6741 | 0.3 | 0.3 | 0.1 | 749000 | 749000 | 248.2 | 0.935 | 700.315 |
| 3D7 | DHA | 3 | sensitive | 0.5 | 113.6 | 80 | 9088 | 0.2 | 0.3 | 0.1 | 1514666.67 | 1514666.667 | 229.5 | 0.907 | 1373.802667 |
| R561H | untreated | 1 | resistant | 2.4 | 152.7 | 80 | 12216 | 0.3 | 0.3 | 0.1 | 1357333.33 | 565555.5556 | 231.7 | 0.967 | 546.8922222 |
| R561H | untreated | 2 | resistant | 2.9 | 98.4 | 110 | 10824 | 0.4 | 0.3 | 0.1 | 902000 | 311034.4828 | 202.1 | 0.813 | 252.8710345 |
| R561H | untreated | 3 | resistant | 2.6 | 42.6 | 160 | 6816 | 0.2 | 0.3 | 0.1 | 1136000 | 436923.0769 | 174.4 | 0.862 | 376.6276923 |
| R561H | DMSO | 1 | resistant | 3 | 20.1 | 210 | 4221 | 0.15 | 0.3 | 0.1 | 938000 | 312666.6667 | 166.8 | 0.785 | 245.4433333 |
| R561H | DMSO | 2 | resistant | 2.8 | 65.6 | 120 | 7872 | 0.6 | 0.3 | 0.1 | 437333.333 | 156190.4762 | 229.1 | 0.87 | 135.8857143 |
| R561H | DMSO | 3 | resistant | 2.6 | 54.9 | 130 | 7137 | 0.15 | 0.3 | 0.1 | 1586000 | 610000 | 225.2 | 0.872 | 531.92 |
| R561H | DHA | 1 | resistant | 0.7 | 56.4 | 110 | 6204 | 0.3 | 0.3 | 0.1 | 689333.333 | 689333.3333 | 229.8 | 0.877 | 604.5453333 |
| R561H | DHA | 2 | resistant | 0.6 | 114.6 | 80 | 9168 | 0.2 | 0.3 | 0.1 | 1528000 | 1528000 | 250.5 | 0.913 | 1395.064 |
| R561H | DHA | 3 | resistant | 0.8 | 65.5 | 100 | 6550 | 0.3 | 0.3 | 0.1 | 727777.778 | 727777.7778 | 220 | 0.896 | 652.0888889 |

kc_count = mean_kcps x duration; kc_total = kc_count/ (df1 x df2 x df3); kc_norm = kc_total/ parasitemia for untreated and DMSO treated conditions; and is equal to kc_total for DHA treated conditions; EVs_count (x10$^{6}$) = kc_norm x Intensity/100; kcps – kilo count per second

**Supplementary Table 2. Analysis of EVs size distribution by parasite lines.**

| Parasite (n = 9) | mean | Standard deviation | min | Q1 | median | Q3 | max |
|---|---|---|---|---|---|---|---|
| PfVps60_KO | 211.0556 | 41.96511 | 160.3 | 179.8 | 205.4 | 228.5 | 281.8 |
| 3D7 | 212.3444 | 30.17549 | 139.7 | 212.0 | 215.6 | 225.0 | 248.2 |
| R561H | 214.4000 | 27.88548 | 166.8 | 202.1 | 225.2 | 229.8 | 250.5 |

**Supplementary Table 3. Post-hoc Dunn's test for the factors: parasite lines (3D7, PfVps60_KO and R561H) and treatment condition (untreated, DMSO-treated, and DHA-treated)**

| Categorical factor | Comparison | Z | P.unadj | P.adj |
|---|---|---|---|---|
| Untreated condition | 3D7 versus R561H | 0.4472136 | 0.6547208 | 0.6547208 |
| | 3D7 versus PfVps60_KO | 1.3416408 | 0.1797125 | 0.5391375 |
| | R561H versus PfVps60_KO | 0.8944272 | 0.3710934 | 0.5566401 |
| DMSO treated condition | 3D7 versus R561H | 0.745356 | 0.45605654 | 0.45605654 |
| | **3D7 versus PfVps60_KO** | **2.385139** | **0.01707266** | **0.05121798*** |
| | R561H versus PfVps60_KO | 1.639783 | 0.10105026 | 0.15157538 |
| DHA treated condition | 3D7 versus R561H | 0.4472136 | 0.6547208 | 0.6547208 |
| | 3D7 versus PfVps60_KO | -1.3416408 | 0.17971249 | 0.2695687 |
| | R561H versus PfVps60_KO | -1.7888544 | 0.07363827 | 0.2209148 |
| 3D7 parasite line | DHA versus DMSO | 0.7453560 | 0.4560565 | 0.6840848 |
| | DHA versus untreated | 1.0434984 | 0.2967175 | 0.8901526 |
| | DMSO versus untreated | 0.2981424 | 0.7655945 5 | 0.7655945 |
| PfVps60_KO parasite line | **DHA versus DMSO** | **2.683282** | **0.007290358** | **0.02187107*** |
| | DHA versus untreated | 1.341641 | 0.179712495 | 0.17971249 |
| | DMSO versus untreated | -1.341641 | 0.179712495 | 0.26956874 |
| R561H parasite line | **DHA versus DMSO** | **2.385139** | **0.01707266** | **0.05121798*** |
| | DHA versus untreated | 1.639783 | 0.10105026 | 0.15157538 |
| | DMSO versus untreated | -0.745356 | 0.45605654 | 0.45605654 |

**Supplementary Table 6. Protein concentration from extracellular vesicles**

| Strain | Treatment | Sample number | Protein concentration (μg/μL) |
|---|---|---|---|
| 3D7 | untreated | 1 | 0.230555558 |
| 3D7 | untreated | 2 | 0.146111105 |
| 3D7 | untreated | 3 | 0.207777781 |
| 3D7 | DMSO | 4 | 0.086296299 |
| 3D7 | DMSO | 5 | 0.662222219 |
| 3D7 | DMSO | 6 | 0.569444444 |
| 3D7 | DHA | 7 | 0.115000006 |
| 3D7 | DHA | 8 | 0.170925912 |
| 3D7 | DHA | 9 | 0.285185174 |
| PfVps60_KO | untreated | 10 | 0.804259232 |
| PfVps60_KO | untreated | 11 | 0.47518519 |
| PfVps60_KO | untreated | 12 | 0.249814826 |
| PfVps60_KO | DMSO | 13 | 0.124814807 |
| PfVps60_KO | DMSO | 14 | 0.250740726 |
| PfVps60_KO | DMSO | 15 | 0.252407407 |
| PfVps60_KO | DHA | 16 | 0.347777778 |
| PfVps60_KO | DHA | 17 | 0.610740749 |
| PfVps60_KO | DHA | 18 | 0.362222218 |
| R561H | untreated | 19 | 0.23037037 |
| R561H | untreated | 20 | 0.242222215 |
| R561H | untreated | 21 | 0.229444442 |
| R561H | DMSO | 22 | 0.272407401 |
| R561H | DMSO | 23 | 0.223148136 |
| R561H | DMSO | 24 | 0.194074066 |
| R561H | DHA | 25 | 0.144444438 |
| R561H | DHA | 26 | 0.153333339 |
| R561H | DHA | 27 | 0.20333333 |

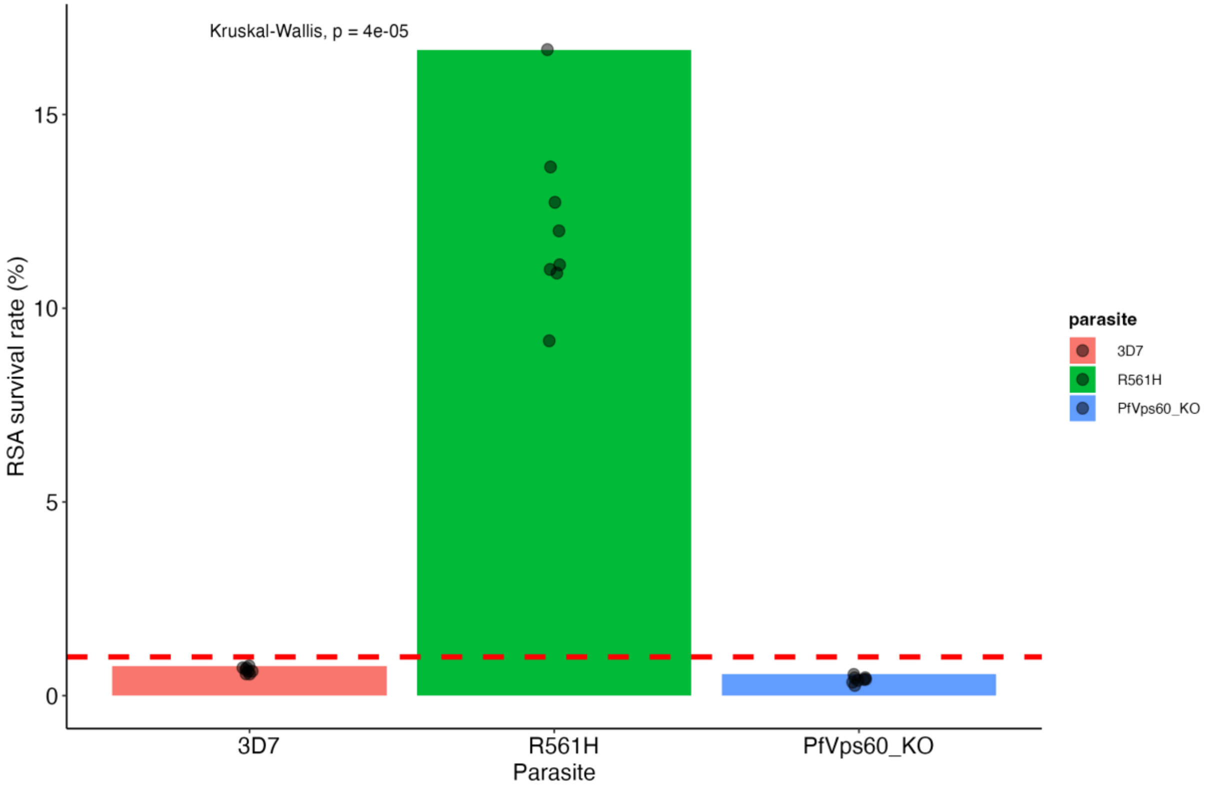


**Supplementary Figure 1.** *In vitro* $RSA_{0-3}$ parasite survival rates (%) to artemisinin (ART) treatment for the *Plasmodium falciparum* strains 3D7, R561H and PfVps60_KO. Each bar represents the mean of triplicate experiments. The red dashed line indicates the 1% survival rate above which a parasite line is characterized as ART resistant.

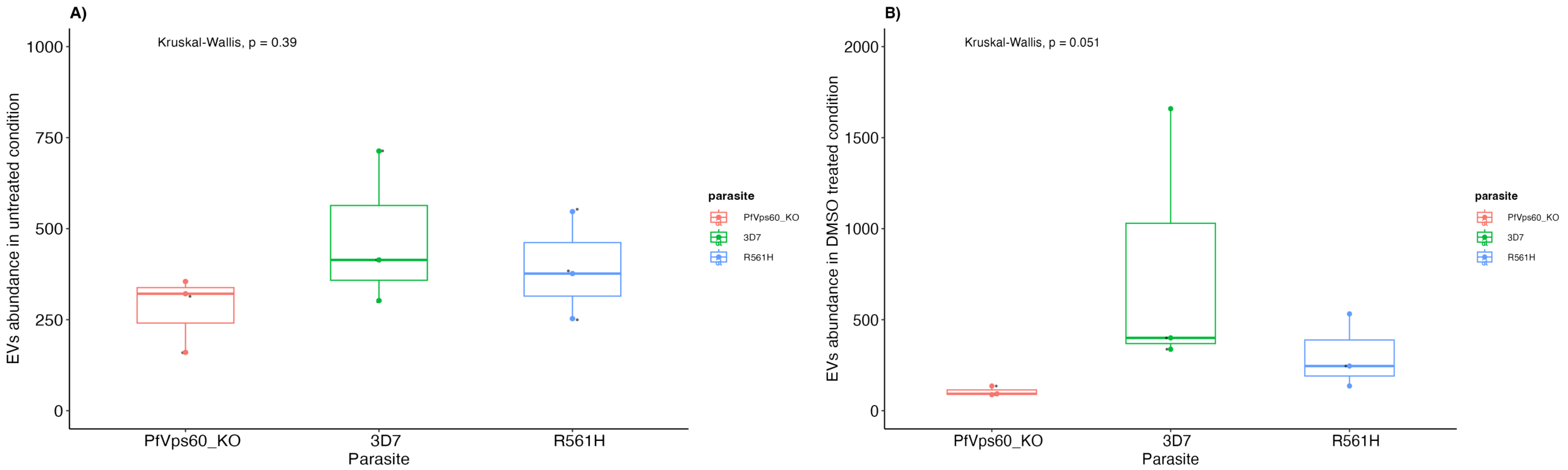


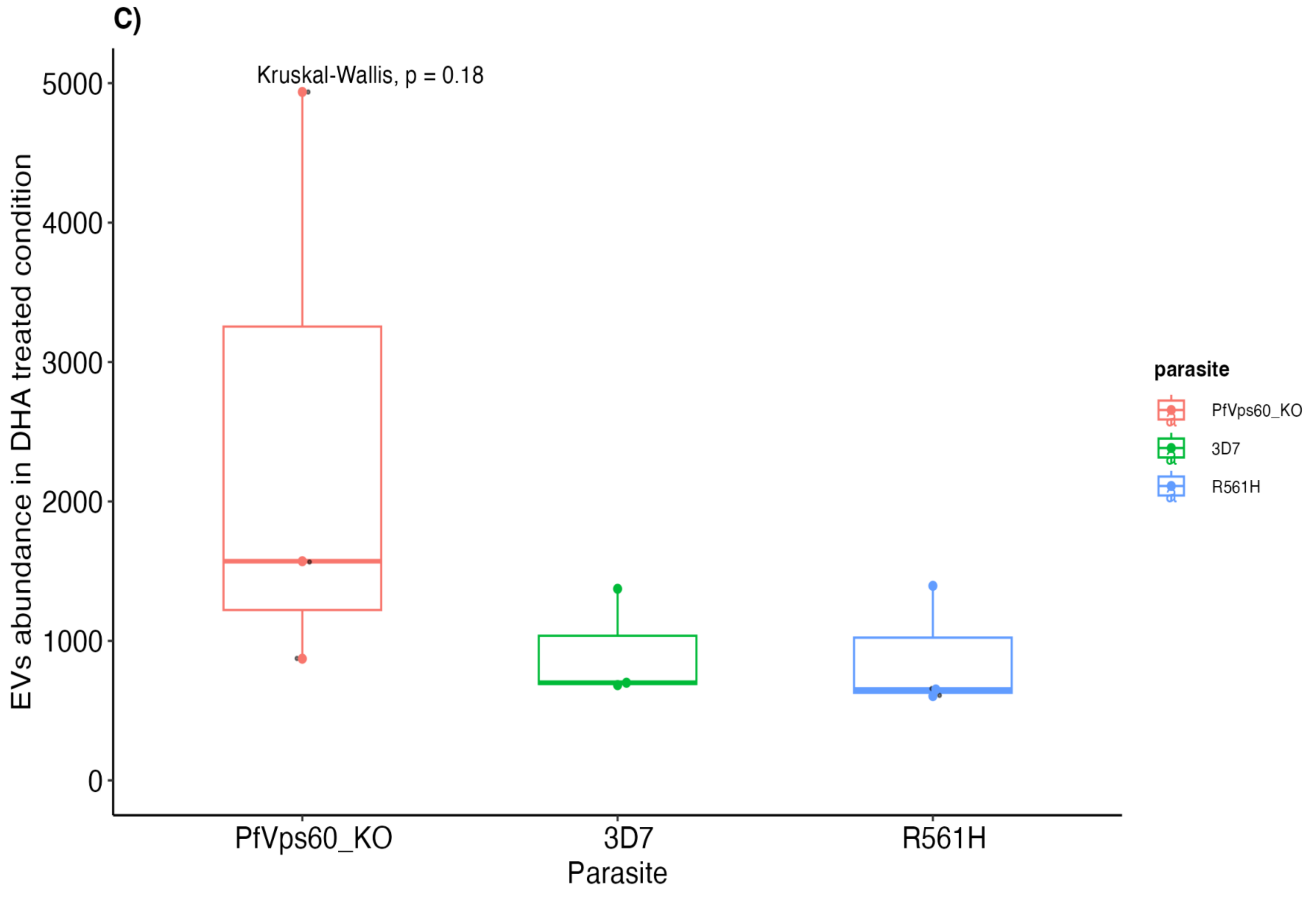


**Supplementary Figure 2** Comparison of extracellular vesicles (EVs) abundance among parasite lines for each of the three conditions studied. The objective was to determine the contribution of each parasite's genetic makeup (predictor or independent factor) to EVs abundance (response/dependent factor). We used the null hypothesis that EVs abundance among the 3 parasite lines was equal and the alternate hypothesis that EVs abundance was not equal. 3D7 is the wild-type artemisinin susceptible parasite line with normal EVs biogenesis pathway. PfVps60_KO was generated from 3D7 by knocking out the *Pfvps60* gene resulting in lower constitutive EVs biogenesis and abundance. R561H is an artemisinin-resistant parasite line CRISPR engineered on a 3D7 background with normal EVs biogenesis pathway. A) In the untreated condition, EVs abundance varied slightly between 3D7 and PfVps60_KO, 3D7 and R561H, and PfVps60_KO and R561H. None of the paired comparisons yielded a p-value to convincingly reject the null hypothesis. B) In the 0.1% DMSO-treated condition, EVs abundance varied between 3D7 and PfVps60_KO, 3D7 and R561H, and PfVps60_KO and R561H, and yielded a p-value = 0.05. C) In the 700nM DHA-treated condition, EVs abundance did not vary between 3D7 and PfVps60_KO, 3D7 and R561H, and PfVps60_KO and R561H and was most abundant in PfVps60_KO.

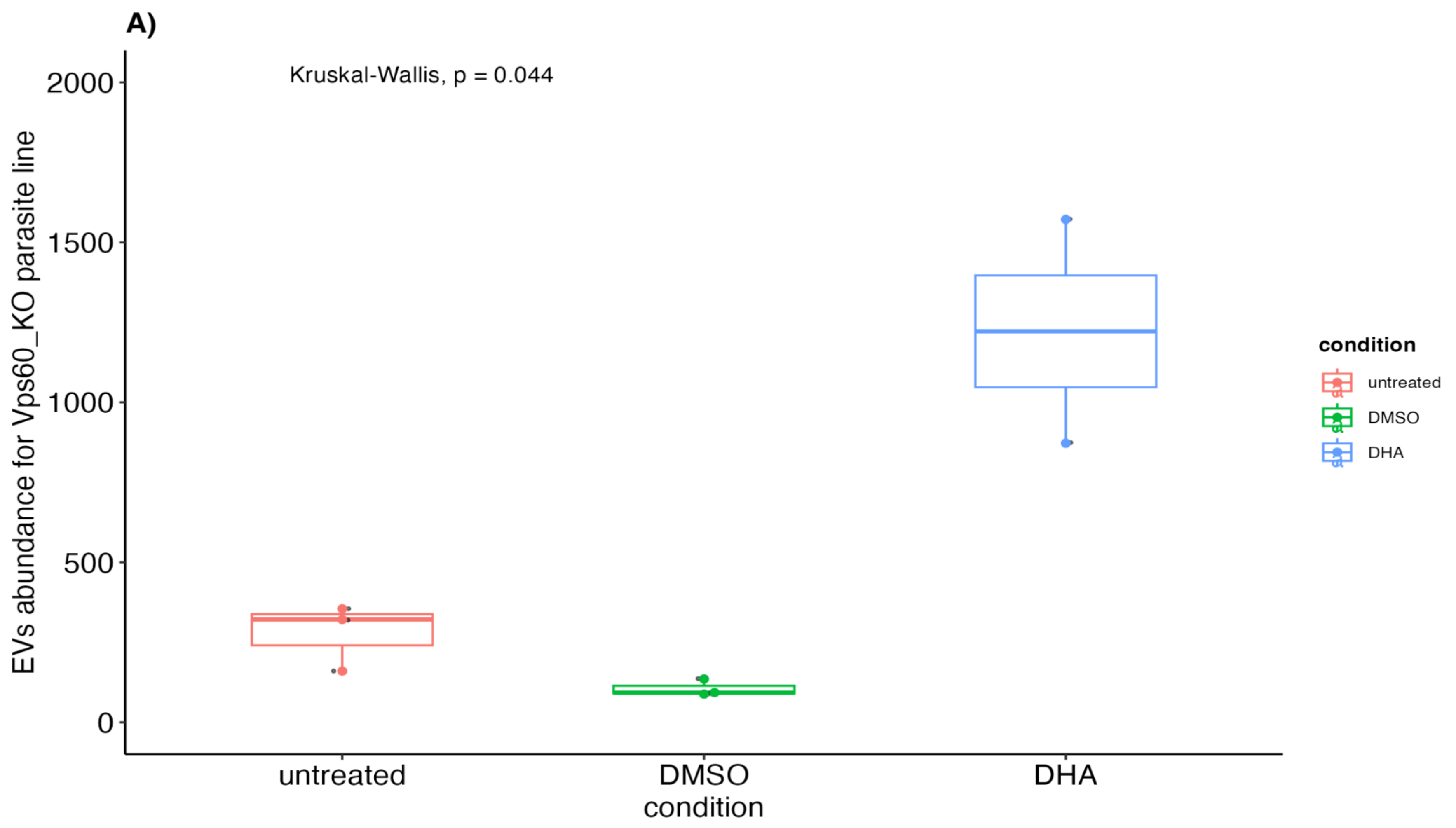


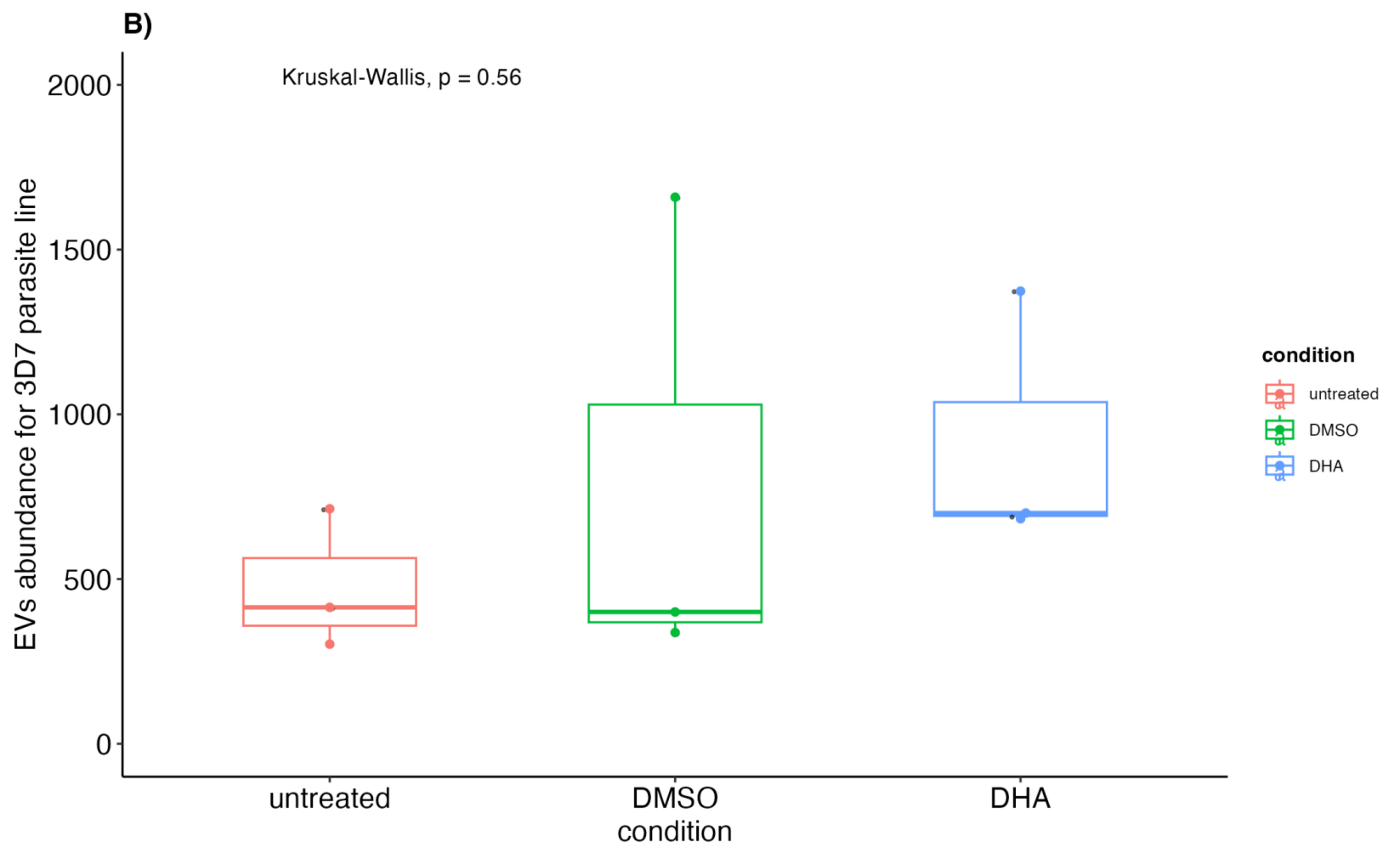


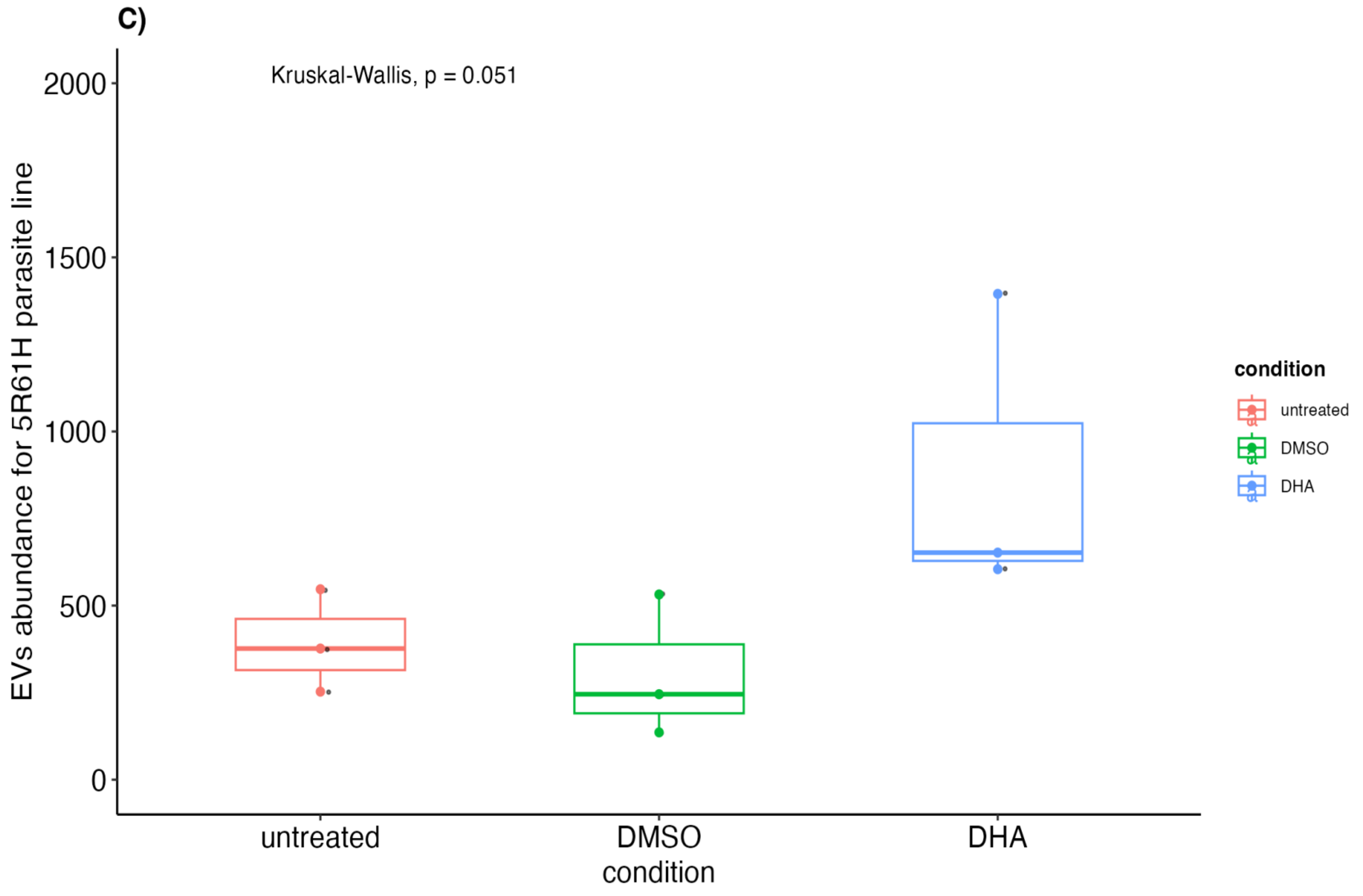


**Supplementary Figure 3.** Comparison of extracellular vesicles (EVs) abundance between treatment conditions for each parasite line studied. The objective was to determine the contribution of each treatment condition (predictor or independent factor) to EVs abundance (response/dependent factor). We used the null hypothesis that EVs abundance among the 3 conditions was equal and the alternate hypothesis that EVs abundance was not equal. 3D7 is the wild-type artemisinin susceptible parasite line with normal EVs biogenesis pathway. PfVps60 was generated from 3D7 by knocking down the Pfvps60 gene resulting in lower constitutive EVs biogenesis and abundance. R561H is an artemisinin-resistant parasite line CRISPR engineered on a 3D7 background with normal EVs biogenesis pathway. A) In the PfVps60_KO parasite, EVs abundance varied between untreated versus DMSO treated, untreated versus DHA treated, and DMSO treated versus DHA treated. Paired comparisons yielded a p-value to convincingly reject the null hypothesis. B) In the 3D7 parasite, EVs abundance varied between untreated versus DMSO treated, untreated versus DHA treated, and DMSO treated versus DHA treated although none of the paired comparisons yielded a p-value to convincingly reject the null hypothesis. C) In the R561H parasite, EVs abundance varied between untreated versus DMSO treated, untreated versus DHA treated, and DMSO treated versus DHA treated and yielded a p-value to marginally reject the null hypothesis.
